# Shoulder Spasticity Treatment With Botulinum Toxin: A Nationwide Cross-Sectional Survey of Clinical Practices

**DOI:** 10.7759/cureus.48493

**Published:** 2023-11-08

**Authors:** Sérgio Pinho, Alexandre Camões-Barbosa, Madjer Hatia, Frederico Moeda, Xavier Melo, João Tocha

**Affiliations:** 1 Physical Medicine and Rehabilitation, Centro Hospitalar de Lisboa Ocidental, Lisbon, PRT; 2 Neurophysiology, Centro Hospitalar Universitário de Lisboa Central, Lisbon, PRT

**Keywords:** botulinum toxin, spasticity, shoulder muscles, clinical practices, survey

## Abstract

Introduction: Upper limb spasticity can be responsible for several complications (e.g., pain, spasms, contractures, deformity, decreased or lost motor control), which can have a negative impact on functional independence and the quality of life of patients. Chemodenervation with botulinum toxin type A (BoNT-A) is a first-line treatment of focal spasticity in the upper limb (UL). However, shoulder muscles were not included in the classical pivotal BoNT-A studies, leaving a knowledge gap regarding the application of intra-muscular BoNT-A in the spasticity management of this anatomical area compared with the arm and forearm.

Materials and methods: We conducted a descriptive cross-sectional nationwide online survey of the current Portuguese clinical practices for BoNT-A injections treating shoulder spasticity. Data were collected regarding the patient’s spasticity cause, shoulder muscles treated, BoNT-A doses, guidance methods used, primary goal domains, treatment effectiveness, adverse effects, and recommendation of adjuvant therapy.

Results: A total of 33 physical medicine and rehabilitation physicians were surveyed. Most of the surveyed doctors (90.91%; n = 30) identified post-stroke spasticity as the major condition for the use of BoNT-A injections in their clinical practice. The most frequently injected muscles for patterns that included shoulder adduction and internal rotation were the pectoralis major (100%; n = 33), subscapularis (93.94%; n = 31), latissimus dorsi (54.55%; n = 18), and teres major (24.24%; n = 8). In patterns including shoulder extension, the posterior deltoid (75.76%; n = 25), the long head of the triceps brachii (66.67%; n = 22), and the latissimus dorsi (48.48%; n = 16) were the most frequently targeted muscles. The primary goals of treatments were improvements in passive function (96.97%; n = 32), pain (84.85%; n = 28), active function (45.45%; n = 15), and range of motion (39.39%; n = 13). The overall impression of therapeutic efficacy was “good” (60.61%; n = 20), and adverse drug reactions were considered “very rare” (84.85%; n = 28) and “mild” (93.94%; n = 31). Ultrasound was used "always" and "most times" in 66.67% (n = 22) of cases. The maximum BoNT-A doses per muscle were lower than those in previously reported studies. Conventional kinesiotherapy was “always” recommended as adjuvant therapy after BoNT-A by 66.67% (n = 22) of physiatrists.

Conclusions: This study provides the first nationwide Portuguese description of "real-life" clinical practices concerning the use of BoNT-A for shoulder spasticity. The selection of goal domains aligned with international results, and the targeted muscles were relatively similar. The use of ultrasound was high, and the maximum BoNT-A doses per muscle were lower than those in other reported clinical practices. The providers reviewed indicated high safety satisfaction with using BoNT-A for shoulder spasticity. Further development of clinical guidelines to standardize practices may be useful.

## Introduction

Spasticity has been classically defined by Lance as a motor disorder characterized by a velocity-dependent increase in tonic stretch reflexes (muscle tone) with exaggerated tendon jerks, resulting from hyperexcitability of the stretch reflex [1]. However, it appears to be a sensory-motor phenomenon and to reflect the involvement of both the sensory input and motor outputs, the Support Program for Assembly of a Database for Spasticity Measurement (SPASM) proposed a new definition: “disordered sensory-motor control, resulting from an upper motor neuron lesion” [2].

Spasticity can be associated with various complications, including pain, spasms, contractures, deformity, and decrease or loss of motor control, which can negatively affect independence in performing activities of daily living, especially in the hygiene and dressing domains, and quality of life, resulting in increased caregiver burden, social isolation, and depression [3-5].

Upper limb (UL) spasticity is reported to affect up to 42.6% of chronic post-stroke patients [6]. Although shoulder spasticity is commonly studied in patients with stroke, it can also be observed in other conditions such as traumatic brain injury, spinal cord injury, multiple sclerosis (MS), and cerebral palsy (CP) [3,5].

Despite the fact that spasticity can affect shoulder muscles, they were not included in classical botulinum toxin type A (BoNT-A) pivotal trials, rendering them a less studied body part compared with the arm and forearm muscles. Nonetheless, shoulder muscles have been included in some BoNT-A clinical trials for UL spasticity [7-11].

Over the last 10 years, multiple studies have demonstrated the effectiveness of BoNT-A as an agent for pain control and improvement of the joint range of motion at the shoulder level. This reflects the usefulness of BoNT-A in attaining a wide variety of patient-centered goals, including passive and active functions, symptoms or impairments, and mobility, such as transfers and walking [3,5,12].

A group of European experts identified the pectoralis major, subscapularis, teres major, latissimus dorsi, biceps brachii, long head of the triceps brachii, and posterior deltoid muscles as frequent targets for intervention in the control of shoulder spasticity [13,14]. However, there appears to be significant variability in muscle selection, formulations, and doses applied, as well as in the guidance methods used by different clinicians [5,15-17].

Kassam et al. conducted a descriptive cross-sectional study in Canada to understand the practices of physicians dedicated to treating spasticity of the shoulder girdle [18].

Currently, there is only one study that describes how Portuguese physicians use BoNT-A to treat shoulder muscle spasticity; however, the published data are based on the clinical practice of a single center [19].

This nationwide study aimed to evaluate the current clinical practices of physiatrists across Portugal, specializing in the treatment of shoulder spasticity in various healthcare settings, including acute care hospitals and rehabilitation centers.

## Materials and methods

Study design 

A descriptive cross-sectional study was conducted at the Centro Hospitalar de Lisboa Ocidental, Lisbon, Portugal. This study was approved by the Centro Hospitalar de Lisboa Ocidental Ethics Committee (approval number: 2379). It was conducted using an online survey among a population of Portuguese physiatrists with experience treating shoulder spasticity.

Survey design 

An online survey was developed using Google Forms® (Google LLC, Mountainview, CA). All data were collected electronically and anonymously.

The questionnaire consisted of multiple-choice questions, four- and five-point Likert scales, and open-ended questions comprising 20 items. These questions included the demographic data of the physicians, patient profiles, targeted shoulder muscles, BoNT-A formulations and doses used, guidance methods, treatment goals and outcomes, and treatment-related adverse effects.

The responses to the questions were both quantitative and qualitative. The survey was designed to take approximately 15-20 minutes to complete. However, no time limit was set for its completion.

Participants 

The study population for this research was Portuguese physicians who are physical medicine and rehabilitation specialists involved in using BoNT-A as a treatment for spasticity. An invitation email was sent to all registered physiatrists affiliated with the Portuguese Society of Physical Medicine and Rehabilitation (SPMFR). This email included a link providing information about our study and access to the questionnaire on Google Forms®. All participants had access to their patients' medical records and could consult them to answer the survey.

This online tool ensured the anonymity of responses by not recording any identifying information about the participants. No incentives were offered for their involvement in the study.

After submission, all questionnaires were carefully reviewed by the first author. Partially answered surveys, defined as those with a response rate of <50% of the 20 questions relevant to this document, were excluded. Descriptive study data were extracted.

Data analysis 

Data were collected and securely stored on the Google Forms® platform and subsequently transferred to a Microsoft Excel® spreadsheet (Microsoft Corp., Redmond, WA). A descriptive analysis of the data was then conducted. For the open-ended responses, a content analysis was performed to report the results later.

## Results

Demographic data of the participants 

A link to access the survey was distributed to 468 registered members of SPMFR. Due to the unavailability of information regarding the number of registered members with experience in using BoNT-A as a treatment for spasticity, an adjusted response rate could not be calculated. However, 33 participants completed the questionnaires, indicating a global response rate of 7.05%. No participants were excluded from the study.

**Figure 1:**
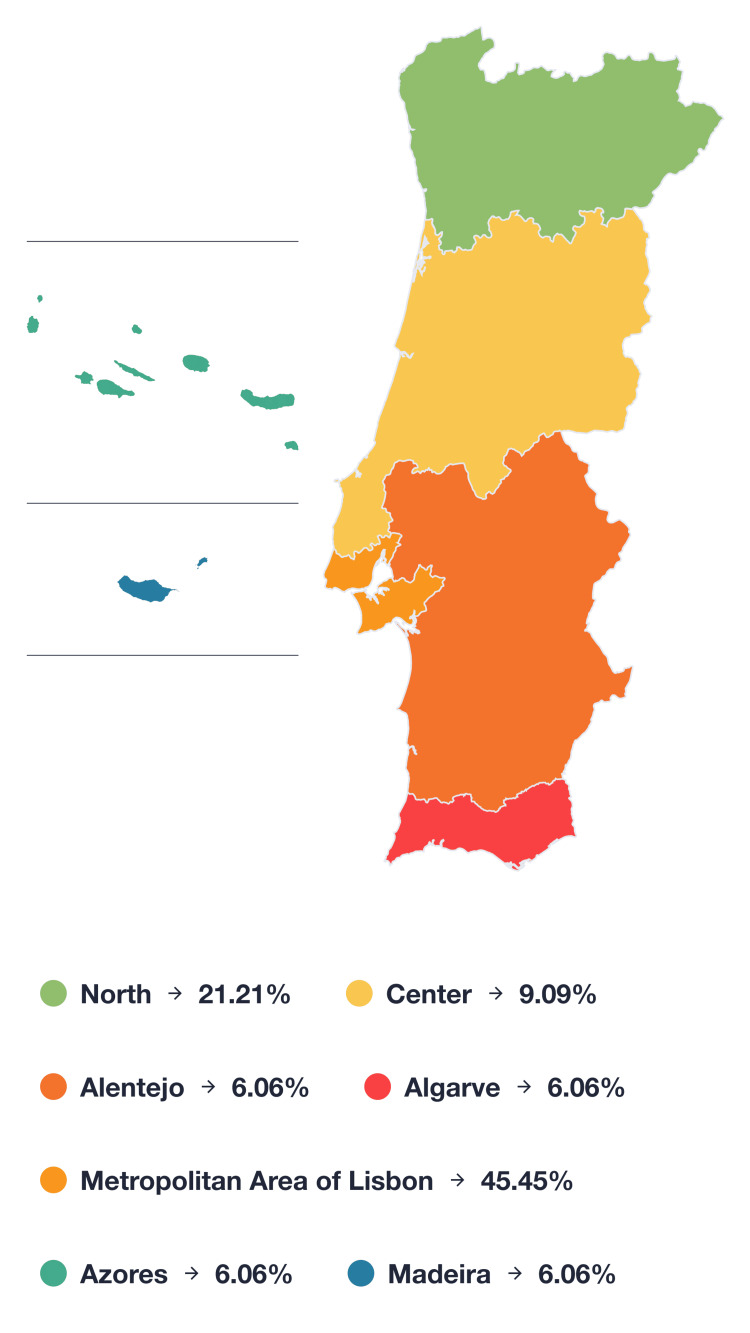
Distribution of surveyed physiatrists by geographical area

This study included Portuguese physiatrists practicing in 22 different acute care hospitals and rehabilitation centers, spanning the northern to southern regions of the country, including the autonomous regions of Madeira and the Azores. The data collected in this study encompassed all geographical areas of Portugal, as depicted in Figure 1.

In terms of experience and working setting, it was found that 42.42% (n = 14) of the participants had experience using intramuscular BoNT-A for ≥10 years. Furthermore, 78.79% (n = 26) of the participants worked in an acute care hospital setting, while the remaining 21.21% (n = 7) were affiliated with rehabilitation centers.

Detailed demographic information on the surveyed physicians is shown in Table 1.

**Table 1:** Demographic data of the surveyed physiatrists BoNT-A: botulinum toxin type A

Questions	Answers	n (%)
Region	North	7 (21.21%)
	Center	3 (9.09%)
	Metropolitan area of Lisbon	15 (45.45%)
	Alentejo	2 (6.06%)
	Algarve	2 (6.06%)
	Azores	2 (6.06%)
	Madeira	2 (6.06%)
Type of unit	Rehabilitation center	7 (21.21%)
	Acute care hospital	26 (78.79%)
What is your experience performing BoNT-A muscle injections for the treatment of spasticity (in years)?	0–9	19 (57.58%)
	10–19	11 (33.33%)
	20–29	3 (9.09%)
	≥30	-

Demographic data of the patients 

The inquiry revealed that the patients, who had spasticity and underwent shoulder muscle BoNT-A injections by the surveyed physiatrists, were mainly males (51.52%; n = 17) and had a predominant age group of 60-69 years (48.48%; n = 16). The most prevalent condition among patients receiving BoNT-A treatment for shoulder spasticity was stroke, representing 90.91% (n = 30), followed by CP (6.06%; n = 2) and MS (3.03%; n = 1).

Among the surveyed physicians, 36.36% (n = 12) believed that >50% of post-stroke patients with shoulder spasticity would benefit from BoNT-A treatment.

Table 2 shows the detailed demographic information of the patients evaluated in the working settings of the surveyed physicians.

**Table 2:** The demographic profile of patients with spasticity evaluated in the working settings of the surveyed physiatrists BoNT-A: botulinum toxin type A

Questions	Answers	n (%)
In your clinical practice, what is the most frequent age group when treating shoulder spasticity with BoNT-A (in years)?	0–18	1 (3.03%)
	19–29	-
	30–39	-
	40–49	-
	50–59	14 (42.42%)
	60–69	16 (48.48%)
	70–79	2 (6.06%)
	≥80	-
In your clinical practice, what is the most prevalent sex group treated with BoNT-A for shoulder muscle spasticity?	Male	17 (51.52%)
	Female	7 (21.21%)
	Similar proportion	9 (27.27%)
In your clinical practice, what are the most commonly treated conditions with BoNT-A?	Stroke	30 (90.91%)
	Traumatic brain injury	-
	Cerebral palsy	2 (6.06%)
	Multiple sclerosis	1 (3.03%)
	Spinal cord injury	-
According to your clinical practice, in which percentage of post-stroke shoulder spasticity patients do you consider to need BoNT-A treatment?	<10%	1 (3.03%)
	10%–20%	4 (12.12%)
	21%–30%	3 (9.09%)
	31%–40%	8 (24.24%)
	41%–50%	5 (15.15%)
	51%–60%	5 (15.15%)
	61%–70%	5 (15.15%)
	71%–80%	1 (3.03%)
	>80%	1 (3.03%)

Clinical practice of physiatrists

Primary Goal Domains

The most commonly identified primary goal domains for BoNT-A shoulder muscle injections for spasticity treatment were passive function (96.97%; n = 32), pain (84.85%; n = 28), active function (45.45%; n = 15), and range of motion (39.39%; n = 13).

Targeted Muscles

The four shoulder muscle groups most frequently targeted with BoNT-A were the internal rotators (100%; n = 33), adductors (100%; n = 33), flexors (72.73%; n = 24), and extensors (21.21%; n = 7). For patients presenting with shoulder adduction and internal rotation, the pectoralis major muscle was identified as the most commonly treated muscle with BoNT-A (100%; n = 33), followed by the subscapularis (93.94%; n = 31), latissimus dorsi (54.55%; n = 18), and teres major (24.24%; n = 8).

Regarding patterns that included shoulder extension, the posterior deltoid muscle was selected as the most frequently injected muscle (75.76%; n = 25), followed by the long head of the triceps brachii (66.67%; n = 22) and latissimus dorsi (48.48%; n = 16).

Botulinum Toxin Type A Formulations

The study participants reported that the most frequently used BoNT-A formulation for shoulder muscle injections was onabotulinumtoxinA (81.82%; n = 27), followed by abobotulinumtoxinA (75.76%; n = 25) and incobotulinumtoxinA (54.55%; n = 18).

Botulinum Toxin Type A Doses

The BoNT-A doses varied for each muscle, depending on the formulation used. Participants were asked to indicate the toxin dose used for each muscle; therefore, typical descriptive statistics could not be calculated. We observed a wide variability in the doses used for each muscle.

Table 3 displays the mean, minimum, and maximum doses for each muscle and BoNT-A formulation according to the study participants.

**Table 3:** Mean, minimum, and maximum doses of BoNT-A formulation for each muscle selected according to the surveyed physiatrists U: units

Muscles	AbobotulinumtoxinA (Dysport®)		OnabotulinumtoxinA (Botox®)		IncobotulinumtoxinA (Xeomin®)	
	Mean U	Minimum–Maximum U	Mean U	Minimum–Maximum U	Mean U	Minimum–Maximum U
Pectoralis major	197	100–300	68	20–100	67	25–120
Pectoralis minor	88	80–100	43	10–60	42	10–60
Teres major	128	50–300	43	10–60	43	10–60
Subscapularis	159	100–300	54	25–80	55	25–90
Latissimus dorsi	198	150–400	64	40–80	63	50–100
Deltoideus (posterior)	143	75–250	50	25–90	51	25–100
Triceps brachii (long head)	196	100–300	59	25–90	58	25–100

Adverse Reactions

The majority of participants (84.85%; n = 28) reported the occurrence of adverse reactions from the BoNT-A application as “very rare,” excluding the effects directly associated with the use of a needle for injection. In instances where adverse reactions did occur, they were reported as "mild" (93.94%; n = 31), characterized by easily tolerable signs and symptoms that could be disregarded by the patient and would fade away when the patient was distracted. Notably, none of the physicians reported pneumothorax as a direct complication resulting from intramuscular BoNT-A injections on the shoulder throughout their careers as specialists in spasticity.

Guidance Methods for BoNT-A Injections

Regarding the frequency of using guided methods for BoNT-A injections in shoulder muscles, 66.67% (n = 22) used an ultrasound “always” and “most times.” The use of electrostimulation and electromyography (EMG) methods was “never” employed in 72.73% (n = 24) and 90.91% (n = 30) of cases, respectively. Finally, anatomical landmarks were used “always” in 30.30% (n = 10), “most times” in 9.09% (n = 3), “sometimes” in 42.42% (n = 14), and “never” in 18.18% (n = 6).

Therapeutic Efficacy

The overall impression of the therapeutic efficacy relating to patients undergoing BoNT-A treatment in the shoulder muscles was rated “very good” in 30.30% (n = 10), “good” in 60.61% (n = 20), and “moderate” in 9.09% (n = 3) of cases. None of the participants considered the perceived treatment efficacy to be “poor.”

Adjuvant Therapy

In response to the question about the use of adjuvant therapy alongside BoNT-A, 66.67% (n = 22) of the participants recommended that conventional kinesiotherapy should be performed “always” after BoNT-A treatment.

The detailed clinical practices of the surveyed physiatrists are shown in Table 4.

**Table 4:** Clinical practices of the surveyed physiatrists BoNT-A: botulinum toxin type A; EMG: electromyography

Questions	Answers		n (%)
In your clinical practice, what are the three most commonly selected primary goal domains in the population of patients undergoing BoNT-A injection of the shoulder muscles for the treatment of spasticity?	Passive function		32 (96.97%)
	Pain		28 (84.85%)
	Active function		15 (45.45%)
	Range of motion		13 (39.39%)
	Muscle tone severity (Modified Ashworth Scale)		4 (12.12%)
	Involuntary movements		2 (6.06%)
	Body image		2 (6.06%)
In your clinical practice, do you use a guidance method when administering BoNT-A injections to the shoulder muscles?	Ultrasound	Never	8 (24.24%)
		Sometimes	3 (9.09%)
		Most times	11 (33.33%)
		Always	11 (33.33%)
	Electrostimulation	Never	24 (72.73%)
		Sometimes	7 (21.21%)
		Most times	2 (6.06%)
		Always	-
	EMG	Never	30 (90.91%)
		Sometimes	2 (6.06%)
		Most times	1 (3.03%)
		Always	-
	Anatomical landmarks	Never	6 (18.18%)
		Sometimes	14 (42.42%)
		Most times	3 (9.09%)
		Always	10 (30.30%)
In your clinical practice, which BoNT-A formulation do you use for shoulder muscle injections when treating spasticity?	OnabotulinumtoxinA		27 (81.82%)
	AbobotulinumtoxinA		25 (75.76%)
	IncobotulinumtoxinA		18 (54.55%)
In your clinical practice, which muscles do you most commonly inject in a spastic pattern of shoulder adduction and internal rotation?	Pectoralis major		33 (100%)
	Subscapularis		31 (93.94%)
	Latissimus dorsi		18 (54.55%)
	Teres major		8 (24.24%)
	Deltoideus		5 (15.15%)
	Pectoralis minor		3 (9.09%)
	Supraspinatus		2 (6.06%)
	Teres minor		1 (3.03%)
	Triceps brachii		1 (3.03%)
	Biceps brachii		1 (3.03%)
	Coracobraquial		1 (3.03%)
	Infraspinatus		-
In your clinical practice, which muscles do you most commonly inject in a spastic pattern that includes shoulder extension?	Deltoideus (posterior)		25 (75.76%)
	Triceps brachii (long head)		22 (66.67%)
	Latissimus dorsi		16 (48.48%)
	Teres major		6 (18.18%)
	Pectoralis major		2 (6.06%)
	Teres minor		-
	Subscapularis		-
	Supraspinatus		-
	Infraspinatus		-
	Pectoralis minor		-
	Biceps brachii		-
	Coracobrachialis		-
In your clinical practice, do you recommend conventional kinesiotherapy after BoNT-A injections to treat spasticity?	Never		-
	Sometimes		1 (3.03%)
	Most times		10 (30.30%)
	Always		22 (66.67%)
In your clinical practice, what is your overall impression of the efficacy in patients undergoing BoNT-A injections in shoulder muscles?	Poor		-
	Moderate		3 (9.09%)
	Good		20 (60.61%)
	Very good		10 (30.30%)
In your clinical practice, how frequent are the adverse reactions after BoNT-A injections in the shoulder muscles?	Very rare		28 (84.85%)
	Rare		5 (15.15%)
	Frequent		-
	Very frequent		-
Which do you consider to be the most common severity grade of adverse reactions after injection of BoNT-A in the shoulder muscles?	Mild (easily tolerable signs and symptoms that could be disregarded by the patient and would fade away when the patient was distracted)		31 (93.94%)
	Moderate (signs and symptoms that cause discomfort and interfere with usual functioning but tolerable. The patient cannot ignore it and it does not disappear when the patient is distracted)		2 (6.06%)
	Serious (signs and symptoms that affect daily life, interrupting it, given its incapacitating nature)		-
	Death		-
In your clinical practice, what was the number of BoNT-A injections used to treat shoulder spasticity that resulted in pneumothorax?	0		33 (100%)
	1		-
	2		-
	≥3		-

## Discussion

It appears that Portuguese physiatrists frequently use BoNT-A for shoulder spasticity, although most of the muscles acting on this joint were not included in pivotal trials and have thus been regarded as off-label for many years. This finding is consistent with that of a previous single-center study [19].

Establishing goals is an essential part of the therapeutic process for patients with spasticity. These goals should be patient-centered, and the patient, their family, or caregiver should be involved in the decision-making regarding what they wish to achieve with BoNT-A treatment within a defined timeframe [20].

Goal domains for treating spasticity have been the subject of several studies. For instance, in the Upper Limb International Spasticity (ULIS)-III study, the most frequently selected goals were related to passive function (56.80%), pain (45.60%), range of motion (35.50%), active function (29.90%), and involuntary movements (29.70%) [21]. Our results are in alignment with those of this study, with passive function (96.97%; n = 32) and pain (84.85%; n = 28) being the most frequently identified primary treatment goal domains. The third most frequently selected domain was active function (45.45%; n = 15), while the range of motion ranked fourth (39.39%; n = 13). It is important to note that 12.12% (n = 4) of the respondents stated that their primary treatment goal was to reduce muscle tone. However, this goal does not align with a patient-centered approach. It is crucial to focus on goals that directly address the needs and well-being of the patient to ensure the most effective and patient-oriented treatment.

Goal Attainment Scaling (GAS) is used to define and assess the achievement of pre-established treatment goals [20]. We asked participating doctors to rate the efficacy of BoNT-A as a treatment for shoulder spasticity and consider the achievement of treatment goals according to the GAS. The results showed that 30.30% (n = 10) and 60.61% (n = 20) rated the perceived efficacy of BoNT-A treatment for shoulder spasticity as “very good” and “good,” respectively, with no reports of its effectiveness being “poor.”

Concerning muscle selection, our findings for Portuguese clinical practices align with those of international studies [18, 22]. For patients with adduction and internal rotation as shoulder spastic patterns, the pectoralis major was consistently identified as the most frequently targeted muscle for injection. Following a decreasing order of frequency, the subscapularis, latissimus dorsi, and teres major were also commonly selected, which is similar to the findings of Kassam et al. and the recently proposed muscles by a group of European spasticity experts [12,13,18]. However, there were some notable differences between our study and those of the Canadian study. The pectoralis minor was the fourth most frequently selected muscle in our study but ranked second among its Canadian counterparts. Conversely, the subscapularis was the second most selected muscle in our study but only the fourth in the ULIS-II study [22]. These variations highlight the nuanced differences in muscle selection among different clinical practices, warranting further investigation and consideration in treatment planning.

The subscapularis is usually considered to be involved in the patterns of spasticity characterized by internal rotation and adduction of the shoulder, and its high frequency of selection in Portugal may be due to the greater accessibility provided with the use of ultrasound. This guiding method allows for real-time needle localization and orientation, increasing the precision of the injection of deeper muscles while also reducing the risk of neurovascular or pulmonary injuries [17,23-26].

Regarding the use of guidance methods for shoulder muscle injections, our study revealed that ultrasound is the most commonly used technique, with 66.67% (n = 22) of surveyed doctors employing it frequently. This indicates a higher percentage of ultrasound use compared with that of the study of Kassam et al. (59.62%) [18]. Despite the relatively recent adoption of ultrasound in the field of spasticity management, it has experienced significant growth, as highlighted by these results [26]. Anatomical landmarks are still frequently used by 39.39% (n = 13), which is slightly higher than reported in the Canadian study (32.69%) [18]. However, we observed that only a small percentage of Portuguese physiatrists currently use electrostimulation (27.27%; n = 9) or EMG (9.09%; n = 3) as guidance methods in most of their injections, which is in contrast with the much higher utilization rates reported in the Canadian study (electrostimulation = 48.08%; EMG = 78.84%) [18]. These differences emphasize the diversity of approaches among clinicians and suggest variations in preferences for guidance methods in shoulder muscle injections. Further research may be needed to understand the factors contributing to these disparities and their impact on clinical outcomes.

The study participants reported using a wide range of doses from the three available formulations of BoNT-A. This variability is expected because the doses of BoNT-A should be individualized for each patient based on the severity of spasticity, specific treatment goals, and other influencing factors [27]. Across all administered BoNT-A formulations, the average dose per muscle was within the recommended ranges and adhered to the safety and efficacy conversion ratio of ≤3:1 (abobotulinumtoxinA:onabotulinumtoxinA) and 1:1 (incobotulinumtoxinA:onabotulinumtoxinA) [28]. This adherence to established guidelines and safety ratios ensures that the treatments are administered with due consideration to both effectiveness and safety, supporting better outcomes for patients with spasticity.

However, for all muscles included in the survey, we found a lower maximum dose reported by our population of Portuguese physiatrists across all BoNT-A formulations when compared with the maximum dose reported by the population of physicians surveyed in the Canadian study [18]. The most significant differences were observed in the maximum dose used for the pectoralis major (-100 U for onabotulinumtoxinA, -200 U for abobotulinumtoxinA, and -80 U for incobotulinumtoxinA), latissimus dorsi (-120 U for onabotulinumtoxinA, -100 U for abobotulinumtoxinA, and -100 U for incobotulinumtoxinA), and deltoid muscle (-110 U for onabotulinumtoxinA, -50 U for abobotulinumtoxinA, and -100 U for incobotulinumtoxinA). These differences appear to highlight a more conservative approach to the maximum dose per muscle among Portuguese physiatrists compared with their Canadian counterparts.

Botulinum toxin type A comes with inherent risks, and there are always potential associated risks during its injection. However, most surveyed doctors emphasized that adverse reactions are very rare (84.85%; n = 28) and usually mild (93.94%; n = 31). 42,42% (n = 14) of the participants reported using BoNT-A for 10-29 years, and none of them reported pneumothorax as a direct complication resulting from BoNT-A shoulder injections throughout their careers.

Of the 33 study participants, 66.67% (n = 22) recommended that conventional kinesiotherapy should “always” be performed after BoNT-A treatment. A single-blind, randomized, controlled trial demonstrated that the use of kinesiotherapy combined with various techniques, such as manual stretching and muscle strengthening, following BoNT-A treatment is associated with benefits in controlling muscle tone and patient satisfaction [29]. The direct effect of intramuscular BoNT-A injection allows for the interruption of the hyperactive stretch reflex and modulation of passive muscle properties. Complementing this with kinesiotherapy may enable a better spread of the drug in the injected muscles and a consequently improved uptake of BoNT-A at the motor plates [29,30].

As a limitation of this study, the possibility of response bias is highlighted, specifically the bias of social desirability, where participants may choose answers that align more with the expected clinical practice than their actual clinical practice. To mitigate this limitation, efforts were made to ensure the anonymity of responses from all participants. However, this may also constitute a limitation of the study, since anonymity does not allow us to ascertain which method was used by each individual to collect medical data and respond to the survey.

Using SPMFR as a distributor of the link added credibility while also targeting physicians who were more likely to be involved in spasticity treatment. The surveyed group of physicians was highly diverse, including doctors from all geographical regions of Portugal who dealt with spasticity stemming from various etiologies and possessed several clinical experiences.

This study offers the first comprehensive nationwide overview of the practices of Portuguese physiatrists engaged in the treatment of shoulder spasticity using BoNT-A, covering both inpatient and outpatient settings. Shedding light on current clinical practices and comparing results internationally may represent a significant advancement in our understanding of the subject and hold the potential to enhance future clinical practices in this field.

## Conclusions

This study revealed that Portuguese physiatrists frequently use BoNT-A for treating shoulder spasticity.

The selection of goal domains aligned with the results from other international studies, showing a preference for passive function, pain, active function, and range of motion. The most frequently selected muscles are the pectoralis major, subscapularis, latissimus dorsi, and teres major. Comparison with Canadian practice shows the subscapularis as a more frequent option and the pectoralis minor as a less frequent option. The use of ultrasound in our study is higher when compared with international results, but there is still a significant percentage of doctors who report using anatomical landmarks in most shoulder treatments. The maximum toxin dose used per muscle is lower than that previously reported, especially for the pectoralis major, latissimus dorsi, and deltoid muscles. The perception of safety when using BoNT-A for shoulder spasticity is very high.

Further development of clinical guidelines to standardize approaches is deemed necessary.
